# Effect of Extender, Storage Time and Temperature on Kinetic Parameters (CASA) on Bull Semen Samples

**DOI:** 10.3390/biology10080806

**Published:** 2021-08-20

**Authors:** Aitor Fernandez-Novo, Sergio Santos-Lopez, Clara Barrajon-Masa, Patricia Mozas, Eduardo de Mercado, Elisa Caceres, Aizic Garrafa, Juan V. Gonzalez-Martin, Natividad Perez-Villalobos, Agustín Oliet, Susana Astiz, Sonia S. Perez-Garnelo

**Affiliations:** 1 Department of Veterinary Medicine, School of Biomedical and Health Sciences, Universidad Europea de Madrid, Madrid, C/Tajo s/n, Villaviciosa de Odón, 28670 Madrid, Spain; aitor.fernandez@universidadeuropea.es (A.F.-N.); natividad.perez@universidadeuropea.es (N.P.-V.); 2 Animal Production Department, Veterinary Faculty, Complutense Univeristy of Madrid, Puerta de Hierro Avenue s/n, 28040 Madrid, Spain; sesantos@ucm.es; 3 National Centre of Selection and Animal Reproduction (CENSYRA) of Colmenar Viejo. Ctra, Colmenar Viejo–Guadalix de la Sierra km 1, Guadalix de la Sierra, 28794 Madrid, Spain; clara.barrajon@madrid.org (C.B.-M.); patricia.mozas@madrid.org (P.M.); agustin.oliet@madrid.org (A.O.); 4 Animal Reproduction Department of National Institute of Agronomic Research (INIA), Puerta de Hierro Avenue s/n, 28040 Madrid, Spain; eduardo.mercado@inia.es (E.d.M.); sgarnelo@inia.es (S.S.P.-G.); 5 Medicine and Surgery Animal Department, Veterinary Faculty, Complutense University of Madrid, Puerta de Hierro Avenue s/n, 28040 Madrid, Spain; elicacer@ucm.es (E.C.); aizicbg@gmail.com (A.G.); juanvi@ucm.es (J.V.G.-M.)

**Keywords:** short-term storage, semen extender, sperm kinetics, computer assisted semen analyses (CASA), BBSE

## Abstract

**Simple Summary:**

The bull accounts for a great part of the economic value of beef herds managed with natural mating, with the Bull Breeding Soundness Evaluation (BBSE) performed in the field becoming essential. Part of the BBSE is the semen-quality evaluation (classical and kinetic parameters) that can be performed in situ (by trained practitioners) or at laboratories, with semen being short-term stored and shipped. The extender used as well as the storage temperature and duration may affect its quality. Thus, our aim was to explore the best conditions to preserve CASA kinetic parameters, to be reliably assessed afterwards. CASA parameters were preserved for up to 4–6 h post-ejaculation, except for AndroMed^®^ in most of the parameters. Motility decreased from 4–6 h up to 24 h, with the best values obtained with BIOXcell^®^ at 5 °C. The rest of kinetic parameters worsened when analyses were performed at 24 h. Therefore, we suggest evaluating seminal quality as soon as possible. However, using BIOXcell^®^, either at 5 °C or room temperature, and INRA96^®^ at room temperature, sperm motility can be reliably evaluated for up to 6 h. These results help to fix adequate protocols for the short-term storage and shipment of bovine semen collected under field conditions.

**Abstract:**

CASA kinetic parameters are often evaluated in a diagnostic centre. How storage conditions affect ejaculates up to evaluation is unclear. We assessed, in 25 commercial bulls electroejaculated in the field, the impact of time until evaluation (0–2 h, 4–6 h, and 24 h post-ejaculation), holding temperature (5 °C vs. room temperature), and extender (AndroMed^®^, BIOXcell^®^ or INRA96^®^) on CASA kinetic parameters. Total and progressive motility, VCL, VAP, VCL, ALH, BCF, STR, LIN, and WOB were assessed. CASA kinetic parameters were preserved for up to 4–6 h post-ejaculation, except for AndroMed^®^. Regardless of extender or temperature, motility decreased from 4–6 h up to 24 h, with the best values obtained with BIOXcell^®^ at 5 °C. Our results suggest that BIOXcell^®^ can preserve sperm motility for up to 6 h, either at 5 °C or room temperature, and also INRA96^®^ at room temperature, with motility assessments and the percentage of the most rapid sperms being the lowest with INRA96^®^ at 5 °C. The kinetic parameters decreased when analyses were performed at 24 h. Therefore, we suggest evaluating seminal quality as soon as possible, before 6 h after collection. These results help to fix adequate protocols for the short-term storage and shipment of bovine semen collected under field conditions.

## 1. Introduction

Since 1974, computer assisted semen analyses (CASA) has been available to objectively assess sperm motion and perform kinetic studies [1]. In its first version, CASA analysed <30 spermatozoa in 4–8 frames. Currently, CASA captures images of 20–1000 sperms in >30 frames and provides comprehensive motion analyses in less than two minutes [1]. The CASA system is based on acquiring consecutive frames from a microscope by means of a simple chip camera [2]. The image is consequently converted to a black/white resolution, exported to the computer, and evaluated by specific software with the CASA module, which is able to analyse these images [3]. In brief, CASA systems project successive digitised images of a sperm suspension onto a detector array; detect objects based on intensity of pixels in a frame or light scatter; and use special software to extract desired information and produce the desired output [4], determining the main kinetic parameters of seminal samples: total and progressive motility, velocities, and sperm movements. Total motility and progressive motility represent the percentage of spermatozoa moving and moving forward, respectively. Other CASA parameters are: curvilinear velocity (VCL, μm/s) which measures the time-averaged velocity along this trajectory; the distance between the first and the last position points in the track segment of a sperm trajectory divided by the total duration of the track is termed straight line velocity (VSL, μm/s); the average of path- and time-velocity along the spermatozoon trajectory is the average path velocity (VAP, μm/s); the deviation from the average path is named amplitude of lateral head displacement (ALH, μm); the frequency that the sperm head moves across the middle plane of the “straightened” is called beat cross frequency (BCF, Hz). Moreover, CASA provides some ratios, such as straightness of the average path (STR; VSL/VAP); linearity of the curvilinear path (LIN; VSL/VCL); and wobble coefficient (WOB; VAP/VCL) [1]. This huge amount of information provided by the computer assisted sperm analyses offers the advantage of reduced bias compared with visual evaluations [5]. Moreover, CASA allows to determine sperm subpopulations: immotile, slow, medium and fast spermatozoa in each field [6]. The sperm subpopulation has been linked to semen quality indicators, such as the activation of motility in the presence of bicarbonate [7], in vitro capacitation [8], cryo-resistance to cell damage [9], or fertility [10,11]. In contrast, there is no common methodology to calculate and study sperm subpopulations [6]. Additionally, it is important to clarify the reasons for the different prevalence of these sperm subpopulations and to assess whether they are of physiological importance [12].

CASA values may allow a more accurate prediction of the potential ‘fertility’ of a bull [13], especially before freezing sperms with fast but nonlinear movements [12], or even in the case of sperms with a reduced motility [11]. However, there is controversy about the predictability of fertility from CASA results [14]. Besides, fertility is variable among bulls, and even between ejaculates from the same bull [12]. Moreover, the handling of ejaculates seems to be crucial to not interfere in quality assessment, and the conditions of semen storage may affect the sperm kinetics, depending on the extender used, the storage temperature, and the storage duration. Previous studies have reported specific effects on kinetic parameters determined with CASA and the relevance of the extender has been previously described [15]. Guimarães and colleagues [16] described that a Tris-egg yolk-glycerol extender had greater cryoprotective action than BIOXcell^®^, inducing better kinetic results (total and progressive motility), even at 2 h and 4 h post-ejaculate. By contrast, Viquez and colleagues [17] observed differences in sperm sub-populations depending on the extender used, revealing that Triladyl^®^ presented relevant differences in kinematic patterns when compared with Tris-egg yolk and OPTIXcell^®^.

In the frame of a broader study of our research group, focusing on semen quality evaluation within the bull breeding soundness evaluation (BBSE) [18], we observed that conditions of short-term storage of bull semen from collection at the farm until delivery to the laboratory (extender, storage time and temperature) affected semen quality, measured in terms of sperm viability, morphology, motility, pH, and microbiological quality. A deeper analysis of the specific kinetic characteristics will help us to understand the link between these conditions and the semen quality impairment and lead to recommendations for semen storage in the field, to best preserve semen quality.

Therefore, our aim in the present study was to mimic different field conditions of short-term storage of bull semen from collection at the farm until delivery to the reference laboratory for analysis (time <2 h, 4–6 h and 24 h; extender: AndroMed^®^, BIOXcell^®^ and INRA96^®^; and holding temperature: refrigeration vs. room temperature) and to measure the effects of that storage on sperm kinetics determined with CASA methodology. We hypothesized that analyses on semen kept under these conditions beyond 2 h would not estimate adequately the cinematic characteristics of bull semen samples, and that if semen needs to be assessed up to 6 h, it would be better to keep it refrigerated, independently of extender. This work is closely related to another study of our group [18], in which we assessed the effects of those conditions in semen quality in terms of viability, morphology, total and progressive motility, pH, and microbiological quality.

## 2. Materials and Methods

### 2.1. Animals and Ejaculate Samples

**Figure 1:**
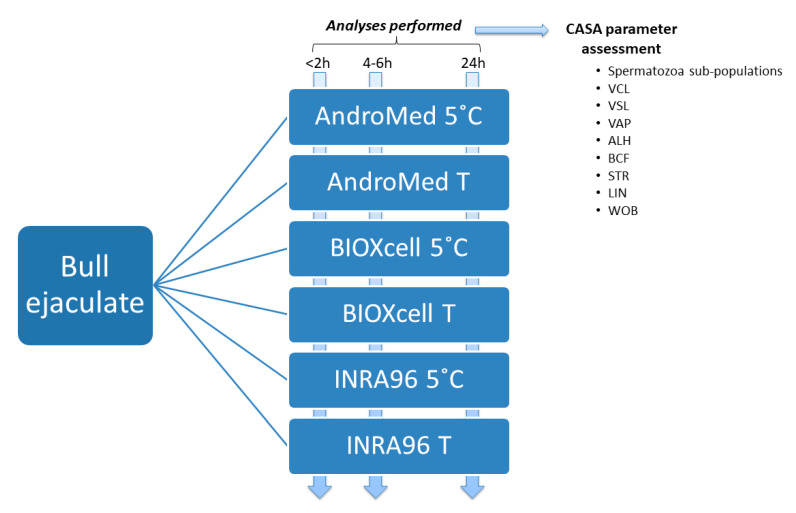
Experimental design (3 × 3 × 2: three extenders, three storage times, by two holding temperatures) and description of laboratory assessments.Abbreviations: T: room temperature; CASA: computer assisted semen analysis; VCL: curvilinear velocity, μm/s; VSL: straight velocity, μm/s; VAP: average path velocity, μm/s; ALH: amplitude of lateral head displacement, μm; BCF: beat cross frequency, Hz; STR: straightness (VSL/VAP); LIN: linearity (VSL/VCL); and WOB: wobble coefficient (VAP/VCL).

This work was carried out concurrently with another study published in the same Journal, as two parts of the same project, using the same ejaculates aliquots [18]. Therefore, the material and methods were all performed as described in that work. In brief, 25 bulls from different commercial Spanish farms were electroejaculated during the routine BBSE of these animals, following advised protocols, such that the study interventions were not considered experimentation on animals and no ethical approval was required. Semen samples were subjected to different conditions that mimicked typical field situations, as described in Figure 1.

A total of 25 bulls from 15 commercial farms from Spain were included in the study. Breeds were Limousine (*n* = 10), Charolais (*n* = 9), cross-breeds (*n* = 3), Blonde D’Aquitania (*n* = 1), Holstein Friesian (*n* = 1) and Spanish Black Iberian Avileña (*n* = 1). This sample size assured an adequate statistical power (0.78–0.85) for the mean semen quality variables assessed. Inclusion criteria for the bulls were: have been apart from the cow herd for more than 15 days; no fever (≤39.0 °C); age between 24–108 months old; no testicular abnormalities revealed with ultrasound scanner exam; scrotal circumference >34 cm and negative testing for bovine viral diarrhea (BVDV), infectious bovine rhinotracheitis (IBR), *Tritrichomonas foetus*, *Campilobacter foetus*, *Besnoitia besnoiti*, and for all officially notifiable diseases specified by the European Union (tuberculosis, brucellosis, peripneumonia, and bovine leucosis).

Animals were restrained in the farm chutes. Bull semen was sampled with electroejaculation, during the routine, yearly Bull Breeding Soundness Evaluation (BBSE). Electroejaculation was performed as previously described [19,20]. All the samples were obtained between May and October 2019. In brief, preputial fur was cut, then the preputium was washed with physiological saline serum and dried with sterile swabs. Transrectal stimulation and faeces removal was performed, before inserting the transrectal probe (75 mm transrectal probe; Electroejaculator Pulsator IV^®^, Lane Manufacturing Inc., Denver, CO, USA) and the automatic program applied. Seminal samples were collected in sterile 15 mL tubes immersed in a 50 mL tube with prewarmed water at 37 °C to avoid temperature shock.

Three factors (extender, holding temperature, and time) were combined to mimic typical field situations to keep and transport/send semen samples from the farms to the diagnostic laboratories. Factor 1: Time-window from collection to CASA-assessment, considering: 0–2 h (“<2 h”: mimicking immediate analyses), 4–6 h (“4–6 h”: mimicking car transportation) and up to 24 h (“24 h”: mimicking postal delivery). Factor 2: Extender, using AndroMed^®^ (A; Minitube, Tiefenbach, Germany; soy bean lecithin-based extender), BIOXcell^®^ (B; IMV Technologies, L’Aigle, France; soy bean lecithin-based extender) and INRA96^®^ (I; IMV Technologies, L’Aigle, France; milk protein-based extender). Factor 3: Holding temperature: refrigeration (5˚C; mimicking the use of standard fridges or temperatures promised by shipping companies) vs. room temperature (23–25 °C). Samples were accordingly identified as AndroMed^®^ 5 °C (A5), AndroMed^®^ room temperature (AT), BIOXcell^®^ 5 °C (B5), BIOXcell^®^ room temperature (BT), INRA96^®^ 5 °C (I5) and INRA96^®^ room temperature (IT), respectively (Figure 1).

Immediately after semen collection, 1:2 aliquots were prepared (1 mL ejaculate; 2 mL extender). If total semen volume did not reach 6 mL six aliquots 1:2 were prepared with 0.5 mL semen with 1 mL extender. No concentration assessment was performed. One aliquot per extender (three aliquots) was kept at 5 °C (“5”) and the other three at room temperature (“T”) up to analysis at the reference laboratory (National Centre of Selection and Animal Reproduction, CENSYRA; Madrid, Spain), at the time windows previously described: <2 h, 4–6 h and 24 h (Figure 1).

### 2.2. Computer Assisted Semen Analyses (CASA)

Aliquots were evaluated with the Computer Assisted Semen Analysis (CASA, Sperm Class Analyser (SCA) Microptic SL^®^; Barcelona, Spain), at the CENSYRA reference laboratory at the time windows, previously described: <2 h, 4–6 h and 24 h. Prior to CASA assessment, samples were warmed up to 37 °C. This system consists of a phase-contrast Nikon Eclipse Ci microscope (Nikon, Japan) attached to a Basler acA780–75 gc digital camera (Basler, Germany) that transmits the images to a computer running the analysis software.

The CASA software was used to evaluate 8 µL of each aliquot of semen (diluted with the same extender until 6 millions of sperm per mL) in a Spermtrack^®^ chamber (20 µm depth) pre-warmed at 37 °C. At least 8 random fields were measured, analysing a minimum of 1000 sperm/sample. Each field includes 50 pictures in 1 s video capture (50 fps).

Average values were calculated for the following parameters based on approximately 1000 spermatozoa and in first instance, the percentage of motile kinematic spermatozoa subpopulations (sp0: static sperms, sp1: slow sperms, sp2: medium and sp3: fast sperms) was assessed. The cut-off values used to differentiate subpopulations were <10 µ/s for sp0, >10 µ/s–< 25 µ/s for sp1, between 25 and 50 µ/s for sp2, and >50 µ/s for sp3. Due to the interest of the fast spermatozoa subpopulation (sp3) [6], the kinetic CASA parameters were studied and results presented globally (including all sperms) and specifically in this subpopulation sp3.

Further kinematic parameters were total and progressive motility (TMot and PMot, respectively), VCL, VSL, VAP, ALH, BCF and the ratios STR, LIN and WOB. Additional configuration parameters for the analysing software were the following: the cell identification area was set at 28–70 μm^2^; sperm with VCL < 20 μm/s were considered immotile, those with VCL 20–60 μm/s were considered slow, those with VCL 60–110 μm/s: medium and VCL > 110 μm/s: fast. STR > 70 was considered to be progressively motile.

### 2.3. Statistical Analyses

Data were analysed using SPSS^®^ version 25 (IBM, Armonk, NY, USA). *p*-values less than or equal to 0.05 were considered significant. Data (all outcomes are continuous variables) are reported as mean ± SD in tables and text and drafted in figures as mean ± SEM.

Outcomes were analysed using a general linear mixed model (GLMM) including the factor bull as a random effect and kept in the model if statistically significant. Double and triple interactions between the studied factors were assessed (extender by holding temperature; extender by time; holding temperature by time and extender by holding temperature by time). Intra-subject tests (Greenhouse–Geisser), intra-subject contrast tests, and inter-subject effect tests were determined. ANOVA for repeated measures analysis was used to examine differences among aliquots within bull at fixed time windows. Potential pairwise correlations were studied by Pearson correlation procedures between all variables at each fixed time window (inter-variables correlations at <2 h; 4–6 h and 24 h) and correlating the values of each studied variable between the different times (intra-variable correlations).

## 3. Results

The analyses of GLMM revealed that the factor “bull” statistically affected all parameters (*p* < 0.01) and was kept in the model. The bulls included in the study were 41.7 ± 23.27 mo. old, their scrotal circumference was 40.3 ± 2.85 cm. and their rectal temperature at electroejaculation 37.8 ± 0.51 °C. The total ejaculate volume averaged 6.7 ± 3.83 mL, ranging between 3 and 15.5 mL, and with the following average and deviation values for each quartile of bulls: Q1, 3.25 ± 0.61; Q2, 4.92 ± 0.20; Q3, 6.08 ± 0.38, and Q4, 12.58 ± 3.11. Additional interesting results of the same ejaculates, e.g., proportion of alive/dead spermatozoa, are described in a previous associated article [18].

### 3.1. Sperm Subpopulations

**Figure 2:**
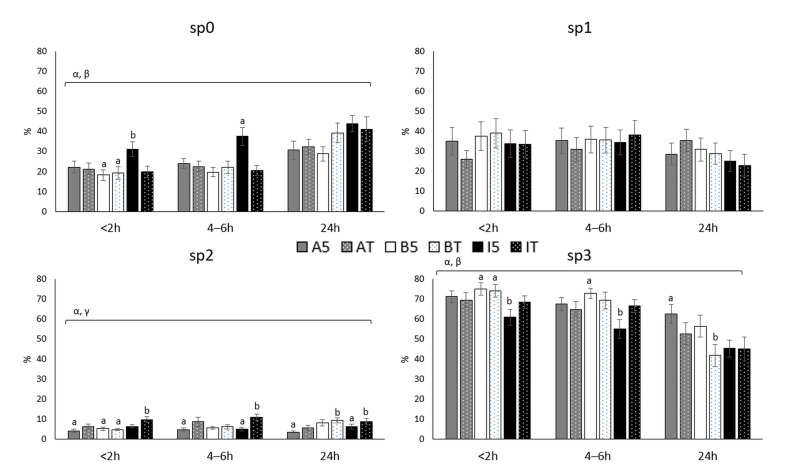
Percentage of bull motile sperm subpopulations (CASA), after storage for three periods (<2, 4–6 and 24 h), in the presence of three extenders (AndroMed^®^, BIOXcell^®^ and INRA96^®^) and two holding temperatures (refrigeration vs. room temperature). Bars with different letters mean statistical significantly different values (*p* < 0.05) among the six combinations extender by holding temperature, at each time window (ANOVA analyses). Square brackets over the bars highlight statistically significant effects (*p* < 0.05; from GLM analyses) of the factors or factors’ interactions (Greek letters), during the time-windows embraced. Abbreviations: CASA: Computer Assisted Semen Analyses; sp0: static subpopulation; sp1: slow subpopulation; sp2: medium speed subpopulation; sp3: fast subpopulation. A: AndroMed^®^; B: BIOXcell^®^; I: INRA96^®^; T: room temperature; 5: refrigeration at 5 °C. < 2h: time of assessment <2 h after collection; 4–6 h: time of assessment 4–6 h after collection; 24 h: time of assessment 24 h after collection; α: interaction temperature by extender; β: interaction temperature by time; γ: interaction extender by time; δ: interaction temperature by extender by time.

The results obtained for motile sperm subpopulations classified by the CASA based on the speed of the sperm movements are depicted in Figure 2.

The fastest sperm subpopulation (subpopulation 3) decreased significantly over time, being affected by the double factor interactions temperature by extender (*p* < 0.01) and temperature by time (*p* < 0.01). The sp3 decreased with time in all samples, with this decrease being similar independently of extender and holding temperature from the time <2 h up to the moment 4–6 h. Moreover, INRA96^®^ 5 °C showed the smallest sp3 subpopulation at both earlier time-windows (60.7 ± 19.04% and 54.8 ± 23.05%, INRA96^®^ 5 °C <2 h and 4–6 h, respectively). However, up to 24 h, the decrease observed in the BIOXcell^®^ at room temperature samples was the steepest (74.0 ± 15.9%, 69.2 ± 20.89%, and 41.8 ± 26.95% for BIOXcell^®^ at room temperature sp3 values at <2 h, 4–6 h and 24 h, respectively), achieving similar levels to those observed in INRA96^®^ samples at 24 h (45.1 ± 20.82% and 44.9 ± 29.91%, INRA96^®^ at 5 °C and room temperature, respectively). AndroMed^®^ 5 °C induced the smallest sp3 reduction over time, keeping the largest sp3 at 24 h (71.1 ± 14.86%, 67.3 ± 16.58% and 62.4 ± 23.08%, <2 h, 4–6 h and 24 h respectively).

### 3.2. Kinetic CASA Parameters

#### 3.2.1. Motility and Velocity Parameters (TMot, PMot, VCL, VAP and VSL)

**Figure 3:**
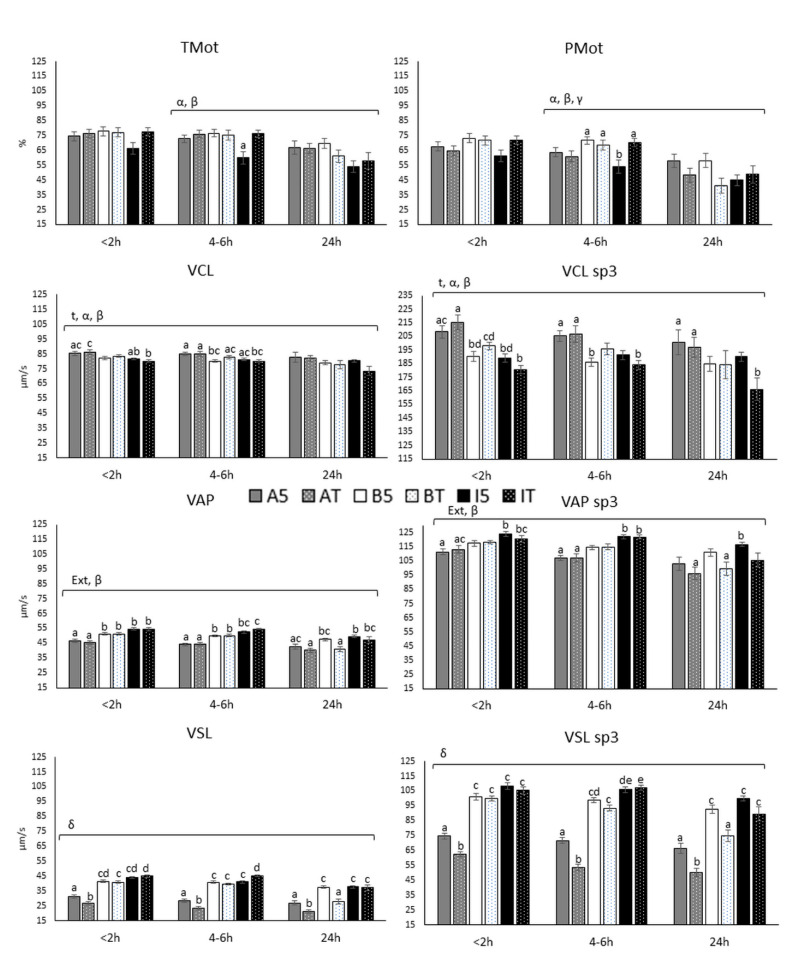
Average bull sperm CASA kinematic motility and velocity parameters of all subpopulations and specifically sp3 subpopulation, at three different time-windows until assessment (<2, 4–6 and 24 h), with three different extenders (AndroMed^®^, BIOXcell^®^ and INRA96^®^) and at two holding temperatures (refrigeration vs. room temperature). Bars with different letters mean statistical significantly different values (*p* < 0.05) among the six combinations extender by holding temperature, at each time window (ANOVA analyses). Square brackets over the bars highlight statistically significant effects (*p* < 0.05; from GLM analyses) of the factors or factors’ interactions (Greek letters), during the time-windows embraced. Abbreviations: CASA: Computer Assisted Semen Analyses; TMot: total motility; PMot: progressive motility; VCL (curvilinear velocity, μm/s); VAP (average path velocity, μm/s); VSL (straight velocity, μm/s); sp3: fast subpopulation. A: AndroMed^®^; B: BIOXcell^®^; I: INRA96^®^; T: room temperature; 5: refrigeration at 5 °C. < 2h: time of assessment <2 h after collection; 4–6 h: time of assessment 4–6 h after collection; 24 h: time of assessment 24 h after collection; α: interaction temperature by extender; β: interaction temperature by time; γ: interaction extender by time; δ: interaction temperature by extender by time.

Results determined with CASA methodology are summarized in Figure 3.

All kinematic parameters decreased with time in the last interval, from 4–6 h up to 24 h after semen collection, independently of extender, with this decrease being more pronounced in samples hold at room temperature, compared to those kept at refrigeration. Significant double interactions temperature by extender (*p* < 0.001) and temperature by time (*p* < 0.01) during the time window from 4–6 h to 24 h affected total motility and progressive motility (Figure 3). The interaction temperature by extender in TMot showed that AndroMed^®^ and BIOXcell^®^ preserved sperm motility differently, depending on the holding temperature (A5 4–6 h: 72.9 ± 12.25%; AT 4–6 h: 76.1 ± 12.40%; A5 24 h: 66.9 ± 22.42%; and AT 24 h: 66.2 ± 16.19%; values for AndroMed^®^ 5 °C and room temperature at 4–6 h and 24 h; vs. B5 4–6 h: 76.7 ± 12.08%; BT 4–6 h: 75.3 ± 15.4%; B5 24 h: 69.9 ± 16.67%; and BT 24 h: 61.1 ± 21.37%; values for BIOXcell^®^ 5 °C and room temperature at 4–6 h and 24 h). BIOXcell^®^ achieved better TMot results when refrigerated than at room temperature, while AndroMed^®^ and INRA96^®^ showed higher values of motility at room temperature than at 5 °C. Regarding the Progressive motility, AndroMed^®^ changed its behaviour when compared to TMot of AndroMed^®^, showing better PMot percentages when hold refrigerated. In both motilities, INRA96^®^ at 5 °C induced the worst results at all time windows (61.4 ± 19.78%, 54.3 ± 22.12% and 44.9 ± 18.88%, <2 h, 4–6 h and 24 h respectively).

Curvilinear velocity was affected significantly by the double interaction temperature by extender (*p* = 0.047) and tended to be affected by the interaction temperature by time (*p* = 0.076). These VCL values did not vary with time up to 4–6 h, independently of extender and holding temperature, but decreased at 24 h (e.g., 85.4 ± 6.12 vs. 82.8 ± 16.32 µm/s for AndroMed^®^ 5 °C at <2 and 24 h respectively). INRA96^®^ at room temperature and BIOXcell^®^ at room temperature showed the most important reductions (80.1 ± 4.23 vs. 73.6 ± 16.16 µm/s for INRA96^®^ room temperature at <2 and 24 h, respectively; 83.5 ± 4.06 vs. 77.6 ± 14.29 µm/s for BIOXcell^®^ room temperature at <2 and 24 h, respectively) compared with the AndroMed^®^ samples (86.1 ± 8.48 vs. 82.00 ± 11.01 µm/s for AndroMed^®^ room temperature at <2 and 24 h respectively). The VCL from the sp3 was affected by the same interactions (temperature by extender; *p* < 0.001 and temperature by time; *p* = 0.046) and the evolution over time in the different groups was consistent with that observed in the global sperm population.

Average path velocity was significantly affected by extender (*p* < 0.001) and by the significant interaction temperature by time (*p* < 0.001), showing that samples kept at room temperature reduced VAP values beyond 4–6 h, especially when using BIOXcell^®^ and INRA96^®^ (50.2 ± 4.18 vs. 40.9 ± 8.38 µm/s; VAP for BIOXcell^®^ room temperature at 4–6 h and 24 h, respectively; 54.7 ± 2.58 vs. 47.0 ± 10.99 µm/s; VAP for INRA96^®^ room temperature at 4–6 h and 24 h, respectively). The extender that preserved VAP differently was AndroMed^®^, independently of temperature and storage time, revealing the lowest VAP values. However, BIOXcell^®^ at room temperature induced the sharpest VAP decrease achieving similar values to AndroMed^®^ at 24 h (40.4 ± 6.87 vs. 40.9 ± 8.38 µm/s; VAP for AndroMed^®^ and BIOXcell^®^ room temperature at and 24 h, respectively). VAP values of sp3 was similarly affected as the VAP in the global sperm population (Figure 3).

Analysis of straight velocity revealed a triple interaction due to extender, temperature, and time (*p* = 0.018), which means that VSL was differently affected by each combination of extender, time, and holding temperature. Globally, AndroMed^®^ was significantly worse than BIOXcell^®^ and INRA96^®^ at all times. In general, lecithin-based extenders kept VSL values better at refrigeration temperature than at room temperature, and independently of extender, values were preserved appropriately between <2 and 4–6 h, with a marked decrease observed from 4–6 h up to 24 h (e. g. A5 4–6 h: 28.5 ± 5.71; AT 4–6 h: 23.7 ± 5.72; A5 24 h: 27.0 ± 6.94; and AT 24 h: 21.3 ± 5.65 µm/s; VSL values for AndroMed^®^ at 4–6 and 24 h at both temperatures). The steepest decrease was observed in semen samples with BIOXcell^®^ and INRA96^®^ at room temperature (39.6 ± 3.32 vs. 28.1 ± 8.18 µm/s, VSL average for BIOXcell^®^ room temperature at 4–6 and 24 h, respectively; 45.3 ± 2.82 vs. 37.2 ± 9.60 µm/s VSL average for INRA96^®^ room temperature at 4–6 and 24 h, respectively). Analysis of sp3 VSL showed a trend of the triple interaction due to extender, temperature, and time (*p* = 0.07), revealing consistent effects with that described for VSL in the global sperm population.

#### 3.2.2. Sperm Movement Parameters (ALH and BCF)

**Figure 4:**
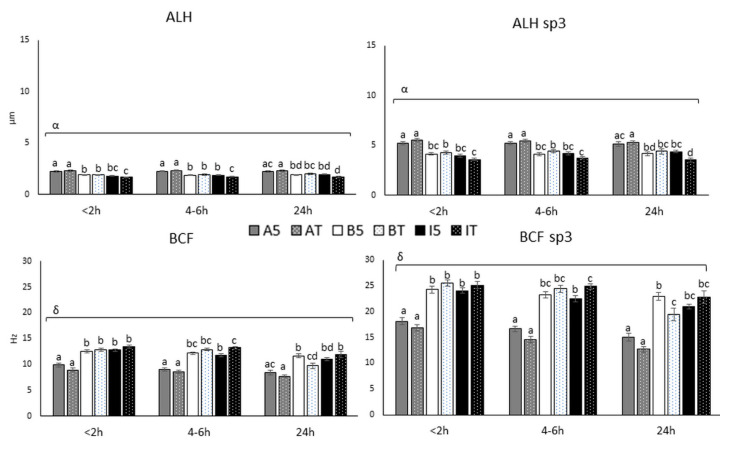
ALH and BCF parameters of all subpopulations and specifically sp3, at three different time-windows until assessment (<2, 4–6 and 24 h), with three different extenders (AndroMed^®^, BIOXcell^®^ and INRA96^®^) and at two holding temperatures (refrigeration vs. room temperature). Bars with different letters mean statistical significantly different values (*p* < 0.05) among the six combinations extender by holding temperature, at each time window (ANOVA analyses). Square brackets over the bars highlight statistically significant effects (*p* < 0.05; from GLM analyses) of the factors or factors’ interactions (Greek letters), during the time-windows embraced. Abbreviations: CASA: Computer Assisted Semen Analyses; ALH: Amplitude of head lateral displacement average; BCF: Beat cross frequency average; sp3: fast subpopulation. A: AndroMed^®^; B: BIOXcell^®^; I: INRA96^®^; T: room temperature; 5: refrigeration at 5 °C. < 2h: time of assessment <2 h after collection; 4–6 h: time of assessment 4–6 h after collection; 24 h: time of assessment 24 h after collection; α: interaction temperature by extender; β: interaction temperature by time; γ: interaction extender by time; δ: interaction temperature by extender by time.

The results obtained for ALH and BCF are depicted in Figure 4.

Amplitude of head lateral displacement average and ALH in sp3 showed an interaction time by extender (*p* < 0.001). AndroMed^®^ samples gave the highest ALH values independently of time and holding temperature (2.3 ± 0.26, 2.3 ± 0.22 and 2.3 ± 0.29 µm for <2 h, 4–6 h and 24 h at room temperature), while INRA96^®^ at room temperature induced the lowest ALH values (1.7 ± 0.22, 1.7 ± 0.19 and 1.7 ± 0.4 µm for <2 h, 4–6 h and 24 h at room temperature). In general, time did not affect ALH (Figure 4). Beat cross frequency average and BCF in sp3 were affected by the triple interaction (*p* = 0.0015 and *p* = 0.0016, respectively), revealing adequately preserved BCF values between <2 and 4–6 h with BIOXcell^®^ and INRA96^®^ independently of holding temperature (12.5 ± 1.56 and 12.2 ± 1.33 Hz for BIOXcell^®^ 5 °C at <2 h and 4–6 h; 12.8 ± 1.07 and 11.78 ± 1.04 Hz for INRA96^®^ 5 °C at <2 h and 4–6 h, respectively). At 24 h a numerical difference between BIOXcell^®^ and INRA96^®^ was observed, and it was statistically different for the sample in BIOXcell^®^ at room temperature (12.9 ± 1.44, 12.9 ± 1.25 and 9.7 ± 2.78 Hz for BIOXcell^®^ room temperature at <2 h, 4–6 h and 24 h, respectively). AndroMed^®^ reported the lowest BCF values independently of time and temperature (8.9 ± 1.70, 8.5 ± 1.71 and 7.7 ± 1.30 Hz for AndroMed^®^ room temperature at <2 h, 4–6 h and 24 h, respectively).

#### 3.2.3. Ratios (STR, LIN and WOB)

**Figure 5:**
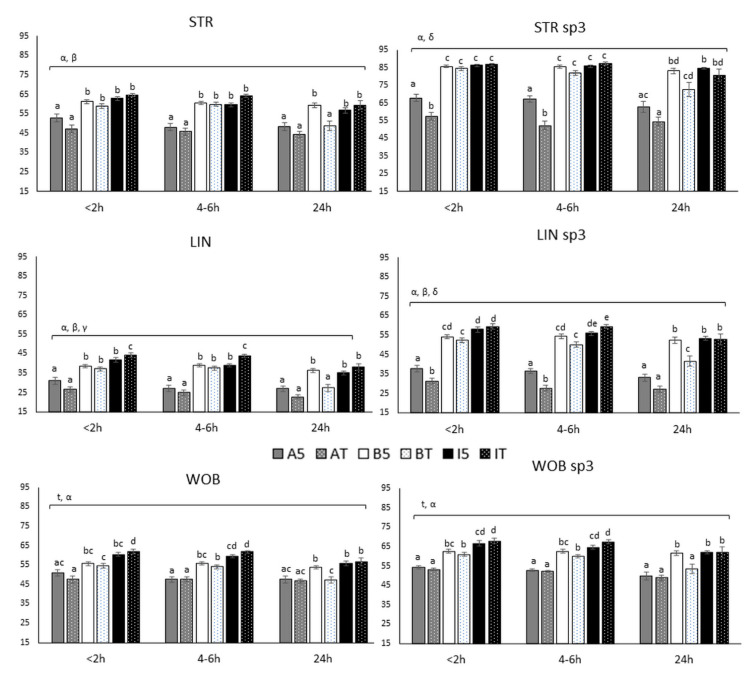
Ratios STR, LIN and WOB of all subpopulations and specifically sp3, at three different time-windows until assessment (<2, 4–6 and 24 h), with three different extenders (AndroMed^®^, BIOXcell^®^ and INRA96^®^) and at two holding temperatures (refrigeration vs. room temperature). Bars with different letters mean statistical significantly different values (*p* < 0.05) among the six combinations extender by holding temperature, at each time window (ANOVA analyses). Square brackets over the bars highlight statistically significant effects (*p* < 0.05; from GLM analyses) of the factors or factors’ interactions (Greek letters), during the time-windows embraced. Abbreviations: CASA: Computer Assisted Semen Analyses; STR: straightness (VSL/VAP); LIN: linearity (VSL/VCL); WOB: wobble (VAP/VCL); sp3: fast subpopulation. A: AndroMed^®^; B: BIOXcell^®^; I: INRA96^®^; T: room temperature; 5: refrigeration at 5 °C. < 2h: time of assessment <2 h after collection; 4–6 h: time of assessment 4–6 h after collection; 24 h: time of assessment 24 h after collection; α: interaction temperature by extender; β: interaction temperature by time; γ: interaction extender by time; δ: interaction temperature by extender by time.

Results of STR, LIN and WOB determined with CASA are summarized in Figure 5.

The analyses of the straightness showed significant interactions temperature by extender (*p* < 0.001) and temperature by time (*p* = 0.035). STR values were kept stable up to 4–6 h independently of the extender, with AndroMed^®^ inducing the lowest STR values at any time and temperature (STR values of 47.3 ± 10.64, 45.9 ± 9.34 and 44.2 ± 8.07 for AndroMed^®^ at room temperature and at the time windows <2, 4–6 and 24 h, respectively). In the time interval from 4–6 h up to 24 h, room temperature samples showed the sharpest STR reduction, revealing important decreases from 4–6 h to 24 h (*p* = 0.06), but also from <2 h to 24 h (*p* = 0.032). This was especially notable for BIOXcell^®^ (STR values: 58.9 ± 6.23, 59.9 ± 5.06 and 48.9 ± 12.08 for BIOXcell^®^ room temperature at the moments <2, 4–6 and 24 h, respectively) and INRA96^®^ (64.6 ± 4.64, 64.2 ± 4.17 and 59.5 ± 11.52 STR values for INRA96^®^ room temperature at <2, 4–6 and 24 h, respectively).

Linearity was affected by all double interactions: temperature by extender (*p <* 0.001), temperature by time (*p =* 0.0194), and extender by time (*p =* 0.03). Linearity results for BIOXcell^®^ and INRA96^®^ were stable up to 4–6 h, with INRA96^®^ at room temperature showing the highest values (44.4 ± 5.63 and 44.1 ± 4.16, LIN INRA96^®^ room temperature at <2 h and 4–6 h, respectively). A decrease was observed at 24 h after semen collection, and it was more notable when room temperature stored, especially for BIOXcell^®^ (37.3 ± 5.43, 37.8 ± 4.74 and 27.6 ± 9.38, values for BIOXcell^®^ room temperature at <2, 4–6 and 24 h, respectively), which reached AndroMed^®^ values at 24 h. Besides, AndroMed^®^ reported the lowest LIN values, independently of time and storage temperature.

The wobble coefficient was significantly affected by the interaction temperature by extender (*p* = 0.001). BIOXcell^®^ and INRA96^®^ kept WOB values stable over time up to 4–6 h, with INRA96^®^ inducing the highest values (62.1 ± 5.93 and 62.0 ± 3.59, WOB values for INRA96^®^ at room temperature at <2 h and 4–6 h, respectively). WOB values decreased with time up to 24 h, decreasing more steeply in samples stored at room temperature, especially for BIOXcell^®^ (54.7 ± 6.31, 54.3 ± 4.90 and 47.4 ± 8.83, WOB values for BIOXcell^®^ room temperature at <2, 4–6 and 24 h, respectively). AndroMed^®^ reported the lowest WOB coefficients independently of time and holding temperature.

The assessment of STR, LIN, and WOB in the subpopulation sp3 showed similar results as those observed in the global sperm population.

### 3.3. Correlation of Different Parameters

All parameters in this study correlated with themselves across the three storage durations, with *r* values > 0.4 (*p* < 0.05), which confirms a general consistency among parameters over time (Figure 3).

There was a positive correlation between motility parameters (total and progressive) and between both motilities with sp3 percentage at any time (Table 1). Moreover, the most intensive correlations among CASA parameters (*r* > 0.8) were found within the same moments of assessment (Table 1).

Correlations between the kinetic parameters VAP, VSL, VCL, ALH, BCF, STR, LIN, and WOB are summarized in Table 2. Again, the most intensive correlations were observed within the same assessment moments.

## 4. Discussion

We conducted a study to evaluate bull semen kinematic parameters, assessed with CASA, during short-term storage. Our results showed that these seminal kinematic parameters were preserved up to 4–6 h post-ejaculation, independently of holding temperature, but were affected by the extender used. The extender AndroMed^®^ led to the lowest values in terms of VAP, VSL, BCF, STR, LIN, and WOB, and the highest ALH values, even at the first moment after collection. Thus, AndroMed^®^ induced a distinctive motility pattern from the first assessment. This may be due to the different composition of the extender; nevertheless, since the exact extender composition is not readily available from the manufactures, interpretation of the results is difficult. All parameters worsened with time, for all extenders and temperatures from 4–6 h to 24 h, with BIOXcell^®^ at 5 °C giving the most conserved values at 24 h indicating that spermatozoa diluted in this extender maintained more efficiently the flagellar structures of the sperms or a better-preserved cell metabolism, both key characteristics for sperm kinematics. The sperm kinematics data reported in the current study could be considered low when compared with values reported previously, which may be due to the differences in the methods used to collect the semen [21], to the differences between beef and dairy bull semen [22], or even to the differences observed among breeds [23]. This last aspect is a variability source for the BBS evaluation, because it is demonstrated the breed relevance in seminal quality.

Computer assisted sperm analyses is an outstanding tool to evaluate bull semen due to its efficacy and enhanced objectivity [5]. However, it is important to specifically describe and fix how semen samples are prepared before and during the CASA assessment, because intra-method discrepancies have been observed [24]. Accordingly, our results showed different effects on the CASA assessments over time, depending on the short-term sperm holding conditions.

The individual bull factor revealed to be important for every single CASA parameter; this result is linked to the previously demonstrated variability of bull ejaculates among bulls from the same and different breeds [25], and even from different ejaculates from one single bull [12]. Due to the lack of homogeneity in breed samples, we cannot conclude that breed would have determined these differences, with this being a limitation of our study. Moreover, due to the scarce number of bulls of a single breed, we could not perform an interbreed comparison. Besides this variability, ejaculates with the largest subpopulations of rapid and progressive sperm are the most resistant to cryopreservation and show the best post-thaw sperm longevity [26]. The subpopulation percentage in seminal samples is also important due to its link to the ability to traverse the barriers of the female reproductive tract to reach the fertilization site [27,28]. Consequently, the optimal conditions to achieve the largest subpopulation of fast sperms, would have a positive effect on fertility [26]. In our study, the ejaculate samples short-term stored (up to 6 h) with BIOXcell^®^ were those with the largest sp3 subpopulation. Beyond 6 h post ejaculate obtention, the soybean lecithin-based extenders at refrigeration better preserved the sp3 percentage. However, AndroMed^®^ did not seem to be as adequate as BIOXcell^®^ for some other CASA kinetic parameters.

Sperm motility decreases with time [29,30]. Consistent with the literature, this research found that total and progressive motility progressively decreased regardless of holding temperature. In refrigerated semen samples, we observed higher motility values using soy lecithin-based extenders than with INRA96^®^. However, Murphy and colleagues [29] described higher progressive motility values than ours when storing samples for up to 24 h at 15 °C with INRA96^®^, with the lack of penetrating cryoprotectant in the extender INRA being probably the reason for this difference among studies, because penetrating cryoprotectants are not required at 15 °C [31].

Velocity parameters measured by CASA can be reliable bull fertility markers. Nagy and colleagues [32] described that VAP was the most relevant and useful parameter, when predicting fertility, vs. VCL and VSL. Other studies have demonstrated that VAP and VSL in fresh semen are positively correlated with motility after thawing [33] and Kathiravan and colleagues [13] revealed a 42.1% of fertility variance due to this two CASA parameters. Thus, a low value of VAP and VSL may predict subfertility of that ejaculate. Based on this premise and our results, the use of BIOXcell^®^ and INRA96^®^ independently of holding temperature, should be preferred to the use of AndroMed^®^ to preserve an ejaculate short-term. On the other hand, in our study, AndroMed^®^ induced statistically higher values for VCL and ALH, revealing that the spermatozoa movement was less straight with AndroMed^®^ than with the other two extenders. Higher ALH values are negatively correlated with fertility, as Farrell and colleagues described [34]. This also accords with the following observation, which showed that STR was significantly lower for AndroMed^®^. These findings induce us to think that the preservation of the potential fertility of ejaculates may be poorer when short-term storing with AndroMed^®^.

Our results corroborate the findings of other studies, which described differences for total motility and VCL when using different extenders (OPTIXcell^®^, Tris-egg yolk-glycerol, AndroMed^®^ and BIOXcell^®^), observing better results with OPTIXcell^®^ than with AndroMed^®^ or BIOXcell^®^, and no statistical difference between both soybean lecthin-based extenders [35]. Murphy and colleagues found also better CASA motility results using OPTIXcell^®^ than AndroMed^®^, although, in terms of fertility (60 days non-return rate), no statistical difference was demonstrated [36]. Another study [37] revealed differences in terms of the velocity, straightness, and linearity of sperms, with OPTIXcell^®^ showing the best values of ALH, BCF, STR, and LIN than those observed with egg-yolk based extenders [38,39]. OPTIXcell^®^ is a protein free and liposomes-based extender, which can explain the differences when compared to egg-yolk based extenders such as Tris-egg yolk-glycerol and to soybean lecithin-based extenders, such as AndroMed^®^ and BIOXcell^®^ [35,40]. However, a recent study shows no difference between AndroMed^®^ and OPTIXcell^®^ in terms of post-thaw sperm quality measured by CASA [41]. Although these previous results can partly explain differences among extenders, we find no clear explanation to the difference observed in our study between AndroMed^®^ and BIOXcell^®^, being both soybean lecithin-based extenders, with AndroMed^®^ inducing a less rectilinear movement of the sperms, when compared to BIOXcell^®^ and INRA96^®^. Most of the previous studies deal with post-thawed semen samples, not with fresh ejaculates as in our work [37]. Viscosity can hinder sperm motility and flagellar movement [42,43] and could be one of the reasons explaining our findings, as Kumar and colleagues revealed [44]. However, we did not perform specific viscosity measurements on the extenders used in the current study. Moreover, it has been described that different concentrations of soybean lecithin in lecithin-based extenders could interfere with seminal quality parameters [45] and, as indicated above, the quantitative composition of these extenders has not been disclosed to the public for commercial reasons.

The holding temperature has been shown to affect seminal kinematic parameters [30]. Our results further support this finding, showing a smaller decrease of all CASA parameters assessed at 24 h when hold at refrigeration vs. at room temperature, although we did not observe any advantage of cooling the semen at 5 °C, when assessed up to 6 h, in terms of CASA kinematic parameters. Similar findings were described previously with egg-yolk based extenders and storing bull semen up to three days [46]. These findings are consistent with a previous research about slow cooling rates prior to freezing semen [30,47]. In the current study, an appropriate cooling ramp was performed, preventing irreversible damage and allowing for the appropriate preservation of the kinematic properties up to 6 h.

In all studied parameters in this study the values correlated with themselves across the three storage durations [47,48], with intense and positive *r* values (>0.4; *p <* 0.05) of the same parameter determined at different time windows. Total motility at <2 h showed an intense positive correlation with PMot evaluated at <2 h and 4–6 h post-ejaculation and with the percentage of fast sperms at <2 h and 4–6 h. We observed positive correlations of VCL at <2 h with ALH at the same evaluation time and between VSL and VAP as well as LIN and BCF. Similarly, Farrell et al. [34] reported significant, intensive correlations between bull fertility (59 d non-return rate) and the following CASA parameters BCF, LIN, VAP, STR, VCL, TMot, and PM. Therefore, computer assisted semen analysis has the potential to more accurately predict fertility than traditional BSE and visual evaluations [34,49], and some CASA kinetic parameters, apart from sperm motility assessment, could be taken into account when analyzing bull ejaculates to exclude in a more reliable way subfertile bulls within a BBSE evaluation, as well as to better estimate bull fertility when thawing seminal samples [50]. In fact, this study revealed that total motility, active cells, beat cross frequency, curvilinear velocity, and amplitude of lateral head displacement measured by CASA have a positive link to bull fertility. However, the current results evidence that the short-term storage conditions for ejaculates (extender, time window, and holding temperature) affect the reliability of these CASA estimations, which should be taken into account in the field when short-term storage of bull semen is required. Further mechanistic studies are worthy to obtain further insights pertaining to the final causes of this variability in the CASA estimations.

## 5. Conclusions

These results reveal that CASA kinematic parameters can be reliably assessed in bull ejaculates up to 6 h after semen collection when using BIOXcell^®^, independently of holding temperature or INRA96^®^ at room temperature. A previous study of our group showed that sperm viability, morphology, motility, pH, and microbiological quality can be kept stable up to 6 h, independently of temperature when using AndroMed^®^ and BIOXcell^®^, or at room temperature in the presence of the extender INRA96^®^. Thus, the best match for a global seminal quality assessment seems to be BIOXcell^®^ at room or refrigeration temperature, up to 6 h post ejaculate collection. If semen samples need to be stored for 6–24 h, BIOXcell^®^ at refrigeration temperature is recommended.

## Figures and Tables

**Table 1:** Pearson coefficients (*r*) for correlations between total and progressive motility and sp3 in bull semen, determined with CASA after three durations of storage in the presence of three semen extenders at two holding temperatures.

	PMotT < 2	PMotT4	PMotT24	Pob3T < 2	Pob3T4	Pob3T24
TMotT < 2	0.947	0.707	0.481	0.922	0.689	0.444
TMotT4	0.756	0.909	0.517	0.759	0.870	0.482
TMotT24	0.519	0.604	0.855	0.570	0.687	0.810
PMotT < 2				0.914	0.674	0.413
PMotT4				0.700	0.898	0.456
PMotT24				0.532	0.619	0.964

Only statistically significant correlations (*p* < 0.05) are shown. Abbreviations: TMot: total motility; PMot: progressive motility; Pob3: spermatic subpopulation type 3 (fast); T < 2: time of assessment < 2 h after collection; T4: time of assessment 4–6 h after collection; T24: time of assessment 24 h after collection.

**Table 2:** Pearson coefficients (*r*) for correlations between kinetic parameters in bull semen, determined with CASA after three durations of storage in the presence of three semen extenders at two holding temperatures.

	VAPT0	VAPT4	VAPT24	ALHT0	ALHT4	ALHT24	LINT0	LINT4	LINT24	BCFT0	BCFT4	BCFT24	STRT0	STRT4	STRT24	WOBT0	WOBT4	WOBT24
VCLT0				0.715	0.52	0.461											−0.438	
VCLT4				0.467	0.714	0.593	−0.491			−0.479			−0.527			−0.440		
VCLT24			0.724			0.87												
VSLT0	0.834	0.558		−0.782	−0.754	−0.561	0.913	0.717	0.476	0.903	0.687	0.491	0.798	0.706		0.786	0.613	
VSLT4	0.577	0.835		−0.691	−0.77	−0.473	0.667	0.943	0.588	0.706	0.879	0.602	0.634	0.897	0.518	0.541	0.814	0.424
VSLT24	0.470	0.569	0.82	−0.407	−0.417		0.431	0.527	0.914	0.429	0.465	0.912		0.482	0.881		0.501	0.799
VAPT0		0.489		−0.532	−0.533		0.797	0.511		0.764	0.463	0.412	0.527	0.482		0.727	0.469	
VAPT4			0.461	−0.579	−0.534		0.455	0.791	0.492	0.45	0.733	0.539		0.666	0.411		0.841	0.432
VAPT24									0.688			0.739			0.653			0.806
ALHT0					0.822	0.609	−0.724	−0.669		−0.714	−0.708	−0.403	−0.522	−0.592		−0.493	−0.664	
ALHT4						0.658	−0.726	−0.778	−0.432	−0.726	−0.797	−0.427	−0.675	−0.725		−0.628	−0.735	
ALHT24							−0.571	−0.465		−0.568	−0.473		−0.543	−0.473		−0.492		
LINT0								0.646	0.451	0.846	0.595	0.414	0.931	0.636		0.926	0.587	
LINT4									0.537	0.658	0.874	0.527	0.610	0.957	0.470	0.530	0.903	
LINT24										0.430	0.480	0.895	0.430	0.508	0.960	0.412	0.505	0.879
BCFT0											0.726	0.481	0.808	0.674		0.776	0.556	
BCFT4												0.533	0.561	0.843	0.422	0.471	0.785	
BCFT24														0.491	0.873		0.494	0.811
STRT0														0.640		0.809	0.507	
STRT4															0.469	0.523	0.791	
STRT24																	0.419	0.821

Only statistically significant correlations (*p* < 0.05) are shown. Abbreviations: VCL: curvilinear velocity (μm/s) VSL: straight line velocity (μm/s); VAP: average path velocity (μm/s); ALH: amplitude of lateral head displacement (μm); LIN: linearity; BCF: beat cross frequency (Hz); STR: straightness; WOB: wobble. T < 2: time of assessment <2 h after collection; T4: time of assessment 4–6 h after collection; T24: time of assessment 24 h after collection.

## Data Availability

Data is contained within the article. Raw data are available on request by the authors.
